# The association between urban–rural disparities and emotional regulation abilities among first-year college students: a statistical indirect effects analysis of psychological and social competence

**DOI:** 10.3389/fpubh.2026.1836842

**Published:** 2026-06-10

**Authors:** Leqin Chen, Qingtong Zhang, YiXia Wang, Chang Liu

**Affiliations:** Shanxi Normal University, Taiyuan, China

**Keywords:** emotional regulation, first-year college students, psychological fitness, social fitness, urban–rural disparities

## Abstract

**Background:**

University students’ emotional regulation abilities are crucial to their mental health. Existing research suggests that urban and rural backgrounds may be associated with emotional regulation among freshmen; however, the underlying mechanisms of this association remain unclear. Drawing on resource conservation theory and the bio-psycho-social model, this study examines whether psychological and social fitness exert statistical indirect effects on the relationship between urban–rural differences and emotional regulation.

**Methods:**

A cross-sectional design was employed to conduct a questionnaire survey of 6,718 first-year students at a comprehensive university. The measurement instruments included the Emotion Regulation Questionnaire, the Emotion Regulation Difficulty Scale, and the Health Adaptation Scale. Control variables included gender, age, single-parent family status, and experience as a left-behind child. Structural Equation Modeling (SEM) was employed to conduct a multiple mediation analysis, and the Bootstrap method was used to test statistical indirect effects, with a focus on the statistical indirect effects of psychological and social fitness. As this study employed a cross-sectional design with a sample drawn from a single university, the statistical indirect effects revealed reflect only statistical associations between variables and do not allow for inferences of causal relationships.

**Results:**

Rural freshmen exhibited significantly greater difficulties in emotional regulation than their urban counterparts, and both psychological and social fitness were significantly lower. Physical fitness was also significantly lower among rural freshmen. Physical, psychological, and social fitness all exhibited significant statistical indirect effects on the urban–rural differences and the three emotional regulation indicators. Multiple mediation analyses revealed that psychological adaptability had the strongest statistical indirect association with difficulties in emotional regulation; social fitness had relatively stronger statistical indirect associations with cognitive reappraisal and expressive suppression; physical fitness showed statistically significant but comparatively smaller indirect effects. The results remained robust after controlling for single-parent family status and experience as a left-behind child. The effect sizes of urban–rural differences across variables ranged from 0.05 to 0.24, falling within the small-effect range; statistical significance should not be equated with practical significance, and urban–rural background itself has limited explanatory power for differences in emotional regulation.

**Conclusion:**

Within the scope of this study, the association between urban–rural disparities and emotional regulation can be explained by the statistical indirect effects of physical, psychological, and social fitness, which serve as the three primary statistical indirect effect pathways. The study indicates that the association between urban–rural differences and emotional regulation is statistically mediated by individuals’ physical, psychological, and social competence resources, providing preliminary exploratory targets for mental health interventions in higher education institutions. However, given the cross-sectional nature of the study, these conclusions require further validation through longitudinal research.

## Introduction

Currently, there is significant attention and concern both domestically and internationally regarding the mental health issues college students face as they develop. In recent years, there have been numerous reports on indicators such as anxiety and depression among college students, and the results show that these indicators have been on a steady upward trend year after year (1). Especially since the outbreak of the COVID-19 pandemic, in order to comply with epidemic prevention and control requirements and reduce the risks associated with large gatherings (2). A study on the mental health of college students in China found that 9.8% of respondents exhibited symptoms of depression and 15.5% exhibited symptoms of anxiety (3). Research indicates that 32 studies involving 93,679 students revealed an overall prevalence of depression of 34.70%. Among this population, individuals with various mental disorders—primarily depression—accounted for 20 to 30% of the total, and more than 80% of the students exhibited psychological problems of significant clinical importance (4).

Current research on emotional regulation and its influencing factors indicates that emotional regulation, as a key mechanism through which individuals cope with environmental stress, is considered a crucial protective factor for maintaining mental health (5). Gross’s three-stage theory of emotion generation divides the emotional process into three components: “trigger,” “evaluation,” and “expression” (6). “Cognition” runs through all three stages, emphasizing that the process of emotion regulation can occur at any stage; that is, individuals can choose appropriate regulation methods based on their own circumstances, thereby reducing negative emotions and enhancing positive experiences, and ultimately achieving the goal of promoting or preventing various physical and mental health outcomes. Therefore, the importance of studying emotion regulation is self-evident (7).

According to American psychologist Erik Erikson, college students are currently facing the crisis of “intimacy versus isolation” (8). This conflict stems from the fact that while individuals seek stable interpersonal relationships, they must simultaneously navigate a constant state of separation. On the one hand, they must integrate into new social circles and build connections with new friends; on the other hand, they must gradually break free from emotional dependence on their parents and establish their own social circles. However, during this process, many students struggle to manage their relationships with others, leading to frequent negative incidents. This can result in imbalances in college students’ physical and mental development, which in turn may trigger various forms of misconduct and even extreme behaviors (9). As can be seen, effective emotional regulation during the freshman year of college helps enhance an individual’s ability to cope with stress, alleviate anxiety, improve stress resilience, maintain a positive state of mind, and effectively prevent the onset of mental health issues, thereby laying a solid foundation for their future development (10). Therefore, this study uses a questionnaire survey to measure and analyze the various emotional responses of students as they enter college, and on this basis, explores effective ways to guide them.

Views on the quality of emotional regulation abilities are primarily divided into two seemingly contradictory hypotheses. The theoretical origins of these hypotheses can be traced back to resource and investment theories and the family and environmental stress model. The resource deprivation hypothesis posits that rural students’ poor emotional regulation abilities are due to a lack of resources (11). The urbanization pressure hypothesis suggests that students in suburban areas face greater psychological stress (12). This contradiction also suggests that urban–rural disparities may not directly affect mental health, but rather exhibit a statistical indirect association through a mediating psychological variable. This study examines psychological and social adaptability as mediating variables to explore their statistical indirect effects on the relationship between urban–rural disparities and emotional regulation.

Engel proposed an integrated bio-psycho-social model to explain the concept of health, defining health and wellbeing as a state in which an individual possesses sufficient physiological, psychological, and social resources to cope with the various challenges of their environment (13). Based on the resource-specificity hypothesis, this study posits that psychological and social competence are the core statistical indirect effect pathways in explaining differences in emotional regulation between urban and rural populations, whereas the statistical indirect effect of physical competence is either insignificant or extremely weak and was therefore excluded from the core analysis. Rather than testing whether such differences exist, this study seeks to determine how psychological and social competence exert statistical indirect effects on the relationship between urban–rural differences and emotional regulation.

In summary, this study aims to use cross-sectional data to explore whether psychological and social adaptability mediate the relationship between urban–rural disparities and emotional regulation through statistical indirect effects. Furthermore, the discussion will carefully distinguish between statistical significance and practical significance to avoid overinterpreting small effect sizes. The study aims to provide preliminary empirical insights into the resource mechanisms underlying urban–rural differences in emotional regulation, thereby laying the groundwork for future longitudinal and intervention studies.

## Theoretical analysis and research hypotheses

### The relationship between emotional regulation and health fitness

The relationship between emotional regulation and psychological and social fitness forms the research framework of this paper. Emotional regulation refers to the process by which individuals regulate their own feelings, thoughts, and actions (14). It is a complex, multidimensional concept that encompasses a wide range of cognitive and behavioral strategies designed to help people alter the intensity, duration, or even the nature of their emotional responses in order to achieve specific goals. The quality of one’s emotional regulation skills directly impacts mental health, significantly influences social functioning, and ultimately determines an individual’s quality of life (15). Having good emotional regulation skills helps people better cope with life’s challenges, maintain a positive and cheerful outlook, and achieve greater success in their interpersonal relationships. Emotional regulation is an indispensable skill in our daily lives.

In this study, psychological fitness is defined as the psychological resilience an individual demonstrates in the face of stressful events, with psychological resilience being its core characteristic (16). Psychological resilience is incorporated into the framework of psychological fitness because it reflects an individual’s internal psychological capacity to persistently cope with challenges, characterized by its expendable, recoverable, and trainable nature (17). Conceptually, the psychological fitness examined in this study is closely related to psychological resilience; however, the term “fitness” is used to emphasize its functional attributes as a dynamic internal resource—it is not a static personality trait, but rather stands alongside physiological energy and social support as a fundamental functional reserve that enables individuals to cope with stress (18, 19). The relationship between psychological fitness and emotional regulation is the most direct and close. Psychological fitness comprises three fundamental components: physical fitness, intellectual factors, and psychological resilience. Psychological resilience refers to an individual’s ability to effectively adapt to and recover from negative experiences, trauma, and stress; it represents the positive emotional regulation resources a person possesses and serves as one of the key foundations for successfully coping with negative stimuli (20). When an individual possesses sufficient psychological resilience as a resource for emotional regulation, they are statistically more likely to demonstrate effective emotional regulation; however, in the absence of sufficient emotional regulation resources, their behavior is statistically associated with greater difficulties in regulation (21). The cognitive adaptation model suggests that psychological resources such as a sense of mastery, self-esteem, and optimism shape an individual’s coping strategies, which in turn can indirectly influence people’s overall assessment of their physical and mental health by improving their psychological wellbeing (22). Research indicates that individuals with high psychological resilience typically employ more constructive and socially acceptable methods of emotional regulation. They may reframe the meaning of situations that trigger negative emotions by altering their perspective on those situations, thereby transforming the original negative emotions. In contrast, individuals with low psychological resilience may resort to more destructive methods of emotional regulation, including emotional suppression or the display of inappropriate behavioral patterns (23). Research shows that both the ability to regulate negative emotions and the ability to regulate positive emotions are closely linked to mental health, and both are also closely related to physical health (24).

In this study, social fitness is defined as the degree of support an individual perceives from their social network, including family and friends, with the core characteristic being the perception of social support (16). The introduction of the concept of social fitness to replace the conventional notion of social support aims to highlight the functional attributes of perceived social support as an external resource. It is not merely a perception of relationships, but rather a reserve of external assistance that individuals can actually draw upon when facing emotional distress. This definition elevates it to a theoretical status on par with psychological fitness and physical fitness, thereby enabling us to examine the unique roles of different dimensions of resilience resources in urban–rural disparities and emotional regulation pathways within an integrated model (25). Socially perceived competence constitutes an exogenous resource for emotional regulation in statistical terms. From a macro perspective, perceived social support refers to an individual’s sense of the likelihood that family members, friends, or others will provide help and support; it plays a significant and indispensable role in emotional regulation (26). In addition to being directly linked to emotional regulation, social support may also exhibit an indirect association through cognitive processes at the statistical level, or function as a behavioral manifestation independent of emotional responses; that is, social support itself serves as a substitute for the suppression of self-expression (27). Research shows that physical activity is statistically associated with higher levels of intergenerational support and social integration, as well as with better physical and mental health. This suggests that social competence is not only an exogenous resource statistically linked to emotional regulation but may also serve as a mediating variable between health behaviors and mental health (28).

The statistical association between physical fitness and emotional regulation is relatively indirect. Individuals who perceive themselves as being in good health tend to have more energy and more stable physiological functioning, which is statistically associated with more effective emotional regulation. Research has found that regular physical exercise is statistically predictive of greater self-control and cognitive reappraisal abilities, as well as higher life satisfaction, suggesting that physical activity is statistically positively associated with more effective emotional regulation (29). A study of athletes found that incorporating mindfulness-based interventions into performance enhancement training significantly improved participants’ cognitive reappraisal and emotional regulation strategies and provided some protection for their mental health (30). Other studies suggest that when individuals experience negative events, possessing positive physical fitness is statistically associated with a reduction in the negative effects of such experiences; it also enables them to better cope with potential future challenges. This process reflects psychological adaptability driven by physiological functions (31).

Based on the above theoretical review, this study proposes that:

*H1*: The levels of psychological and social fitness among rural freshmen are significantly lower than those of urban freshmen, and they experience significantly greater difficulty with emotional regulation than urban freshmen.

### Indirect effects of psychological and social fitness

Resource conservation theory further explains that individuals strive to acquire, retain, and protect resources that are valuable to them (32). Psychological and social fitness are, to a certain extent, important mental health resources; individuals with ample psychological resources are statistically associated with adaptive emotional regulation, while those lacking such resources are statistically associated with difficulties in negative emotional regulation (33). Place of residence, as a sociodemographic variable, is statistically associated with levels of health-related fitness and psychological functioning. Compared to urban living, rural living conditions are relatively harsh; young people who grow up in such environments report statistically lower levels of health-related fitness resources and exhibit statistically more pronounced stress responses when faced with pressure (34). Young people who have grown up in relatively affluent urban environments report statistically higher levels of health and fitness resources and are statistically more likely to seek help promptly when facing difficulties (35).

Existing research has confirmed the existence of statistical indirect effects from the perspective of the resource–adaptation–health pathway. A study by Peeters et al. (36) on patients with rheumatoid arthritis demonstrated that the relationship between adaptive capacity and health can generate statistical indirect effects through mental health. This finding suggests that the statistical association between resilience and health status only becomes apparent when the target variable is mental health. As a distal background variable, place of residence may need to be mediated through a proximal variable such as health-related fitness in order to reveal its statistical association.

Similar statistical mediating effects are also observed in the field of health behaviors. Research by Wiedmaier-Barros et al. (37) indicates that there is a positive correlation between moderate-to-vigorous physical activity and health-related quality of life, and that this relationship can be mediated by muscle strength and body composition. Although this study used physiological indicators as mediating variables, the behavior–physiological resources–health pathway it describes is consistent with the social context–psychological resources–emotional regulation pathway outlined in this paper. Another study conducted with adults demonstrated a more indirect relationship: regular physical exercise may be associated with an individual’s cognitive and emotional regulation abilities, which in turn are statistically linked to the effects of physical activity levels and social support levels on life satisfaction (29). This also suggests that even health behaviors that seem quite distant may be linked to emotional regulation, and thus to an individual’s psychological wellbeing.

**Figure 1:**
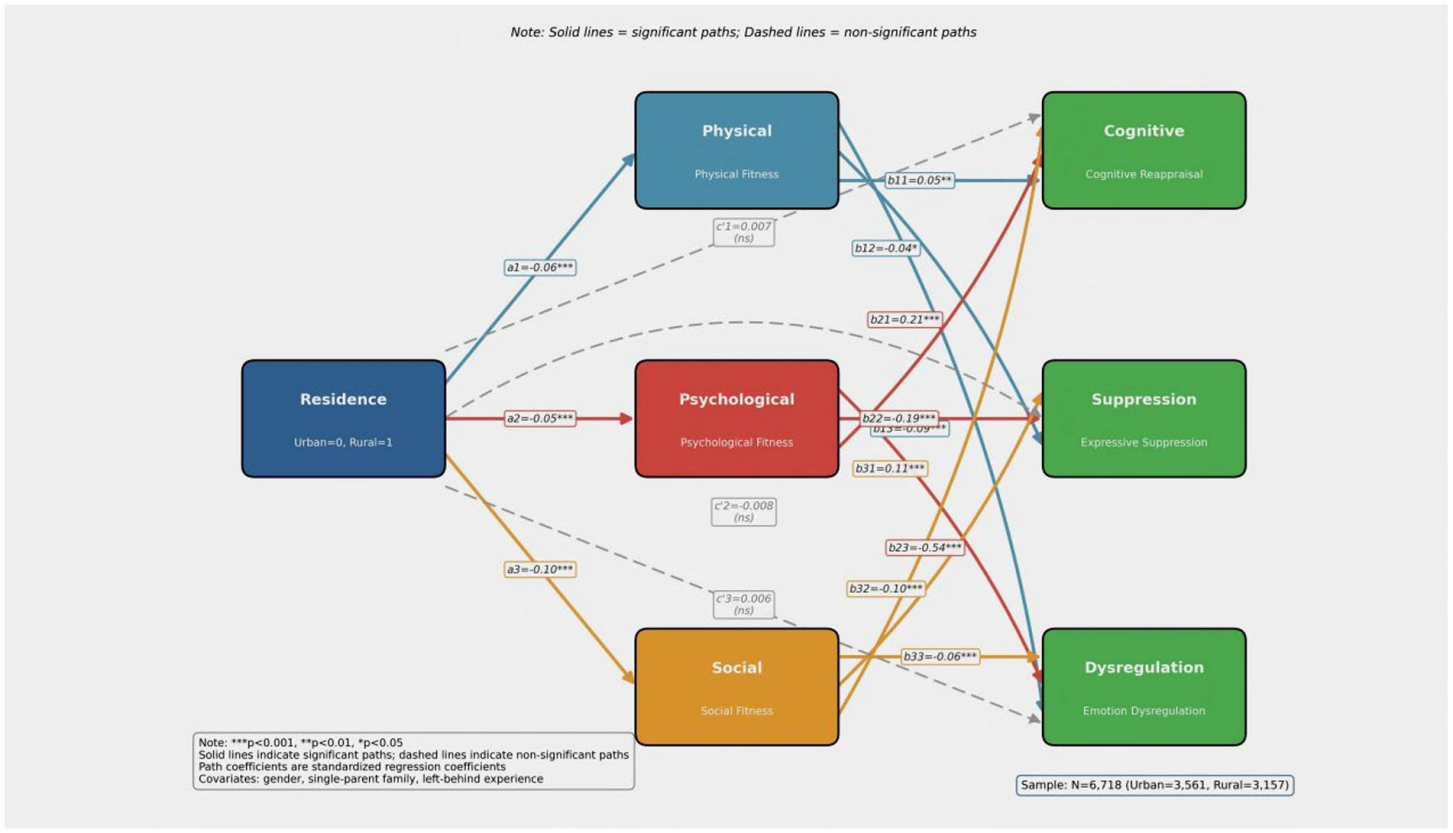
Conceptual diagram of the mediation model. ***p < 0.001, **p < 0.01, *p < 0.05. Solid lines = significant paths; dashed lines = non-significant paths. All path coefficients are standardized. Control variables: gender, age, single-parent family, and left-behind child experience.

Once health-related fitness is controlled for, the direct predictive effect of urban–rural differences on emotional regulation diminishes; it can therefore be inferred that psychological and social fitness may serve as statistical mediators of the relationship between urban–rural differences and emotional regulation (38). In a recent study of athletes, recreational exercisers, and non-exercisers, physical activity was found to have a statistically significant inverse association with psychological distress through the indirect effects of cognitive reappraisal and expressive suppression (39). Based on the resource-specificity hypothesis, this study further proposes that different types of health resources may have distinct statistical indirect effects on urban–rural disparities and emotional regulation. Psychological resilience is more closely statistically associated with internal cognitive restructuring and the maintenance of resilience, while social resilience is more closely statistically associated with alternative pathways of emotional regulation facilitated by external support networks (Figure 1).

Based on the above theoretical analysis, this study proposes that:

*H2*: Both psychological and social fitness have significant statistical indirect effects on the relationship between urban-rural differences and emotional regulation.

*H2a*: Urban-rural differences statistically predict difficulties in emotional regulation, cognitive reappraisal, and expression suppression through the statistical indirect effect pathway of psychological fitness.

*H2b*: There are differences in the statistical indirect effects of psychological and social fitness, such that the statistical indirect effect of psychological fitness on difficulties in emotional regulation is stronger, while the statistical indirect effect of social fitness on cognitive reappraisal and expression suppression is stronger.

*H3*: The statistical indirect effects of physical fitness are significant but comparatively weaker than those of psychological and social fitness.

## Methods

### Research design and study population

This study employed a cross-sectional design and conducted a questionnaire survey in September 2025 among all first-year undergraduate students enrolled in 2025 at a comprehensive university. This timing was chosen because adaptation during the early stages of enrollment serves as a predictor of mental health over the subsequent 4 years; furthermore, weeks 1 through 6 represent a critical period during which new students initially complete their adjustment to university life, while not yet being significantly affected by academic pressures such as midterm exams.

This study employed a stratified cluster sampling method covering all departments across the university. While this approach ensures broad coverage, the reliance on voluntary participation within classes may introduce selection bias, and the external validity of the findings requires further verification. A total of 7,200 questionnaires were distributed, with 6,718 valid responses returned, resulting in a valid response rate of 93.3%. Inclusion criteria were as follows: (1) first-year undergraduate students in the Class of 2025; (2) Age ≥ 16 years; (3) Voluntary participation and completion of informed consent. Exclusion criteria were: (1) Questionnaire completion time shorter than 5 min or longer than 60 min (based on the reasonable response time range determined in the pilot study); (2) Presence of systematic response patterns (e.g., selecting the same option for 10 consecutive questions); (3) Failure to complete the measurement of core variables.

There were 4,689 females (69.8%) and 2,029 males (30.2%); 3,561 had urban household registration (53.3%) and 3,157 had rural household registration (46.7%); 712 came from single-parent families (10.6%); and 1,583 had experienced being left-behind children (23.6%). Coding of key variables, urban rural was coded as 1 = urban, 2 = rural; gender was coded as 1 = male, 2 = female; single-parent family was coded as 1 = no, 2 = yes; left-behind child experience was coded as 1 = no, 2 = yes. Age was treated as a continuous variable.

### Research procedure

The questionnaire was distributed via the Wenjuanxing online platform and was completed anonymously to minimize social desirability bias. Students received invitation links through their class chat groups and could complete the survey at their convenience on either a mobile device or a computer; the average completion time was 18.3 ± 5.7 min. The questionnaire’s homepage clearly outlined the research objectives, the principle of voluntary participation, the right to withdraw at any time, and the commitment to data confidentiality. Prior to distribution, this study had obtained approval from the Ethics Committee (2024–0321), and all students read and confirmed the informed consent form before responding.

### Measurement tools

Urban–rural differences were measured using a single item: “Where did you primarily live before graduating from high school?” The response options were: 1 = urban (including cities and county seats), 2 = rural. In subsequent analyses, urban was coded as 0 and rural as 1 to facilitate the interpretation of regression coefficients. The use of a single-item measure has limitations; it struggles to capture the continuity and complexity of urban–rural backgrounds and may underestimate the effects of urban–rural differences, potentially affecting statistical inferences from the model. However, given that the urban–rural binary household registration system remains one of the primary dimensions of social structure in China, and that this item has been widely used in previous studies of this type to effectively reflect the main variations between urban and rural groups, this simplified measurement method is retained in the present study. The Cronbach’s *α* coefficient for the Social Adaptation Scale was 0.685, which is at the acceptable threshold; this may introduce measurement error and should be taken into account when interpreting subsequent results.

Emotional regulation was assessed using two sets of tools to provide a multidimensional evaluation. The Emotional Regulation Questionnaire developed by Gross et al. (40) was used. This scale consists of 10 items, including cognitive reappraisal (6 items) and expressive inhibition (4 items) (41). The two dimensions were measured using a 7-point Likert scale (1 = Strongly Disagree, 7 = Strongly Agree). In this study, the Cronbach’s *α* coefficient for the Cognitive Reappraisal dimension was 0.86, and for the Expressive Inhibition dimension, it was 0.81, indicating that both dimensions possess good internal consistency reliability. The Emotional Regulation Difficulty Scale, originally developed by Gratz et al. (41) and revised by Chinese researchers, was used. The scale consists of 16 items covering six dimensions: non-acceptance of emotional reactions, difficulties with goal-directed behavior, difficulties with impulse control, lack of emotional awareness, limited regulation strategies, and lack of emotional clarity. The scale uses a 6-point rating scale, with higher total scores indicating more severe difficulties in emotional regulation. In this study, the Cronbach’s *α* coefficient for the total scale was 0.94, and the Cronbach’s α coefficients for the subscales ranged from 0.79 to 0.88, indicating good reliability. The results of confirmatory factor analysis showed that the six-factor model had good fit indices (*χ*^2^/df = 2.96, CFI = 0.92, TLI = 0.91, RMSEA = 0.062, SRMR = 0.055), indicating that the scale possesses good construct validity in this sample and laying the foundation for subsequent structural equation modeling analysis.

This study used the Health and Fitness Scale to measure individuals’ psychological and social fitness (42). Originally named the Health Fitness Scale, this instrument comprises three subscales: physical fitness, mental fitness, and social fitness. In line with the research focus, this study primarily utilized the mental fitness and social fitness dimensions, while the physical fitness dimension was not included in the model as a core variable. Confirmatory factor analysis was conducted to examine the fit of the two-dimensional model among a sample of Chinese college students. The results indicated good fit indices: *χ*^2^/df = 3.52, CFI = 0.95, TLI = 0.94, RMSEA = 0.055, SRMR = 0.040. These results support the two-dimensional structure, making it suitable for latent variable analysis in structural equation modeling. The psychological fitness scale used the Connor-Davidson Psychological Resilience Scale (Short Form), consisting of 10 items rated on a 5-point scale (1 = Never, 5 = Always). To some extent, this scale reflects the attitudes and coping strategies exhibited by participants in response to life events, as well as their level of expectation regarding future life. In this study, the Cronbach’s *α* coefficient for this subscale was 0.723. The social fitness scale used the Social Support Perception Scale revised by Li Hong et al., consisting of 12 items and employing a 7-point rating scale (1 = strongly disagree, 7 = strongly agree). This scale is used to measure the degree of perceived social support from family, friends, and other sources, as well as the individual’s subjective evaluation of its value. In this study, the Cronbach’s alpha coefficient for this subscale was 0.685, which is at an acceptable threshold; however, its relatively low reliability may introduce measurement error, and caution is required when interpreting the results.

Since all variables in this study were measured using self-report scales, common method bias may be a concern. An unrotated exploratory factor analysis was conducted on all measurement items using Harman’s one-factor test. The results showed that there were 12 factors with eigenvalues >1, and the first factor explained 18.63% of the variance, which is below the critical threshold of 40%. This indicates that there is no serious common method bias in this study.

To control for the potential influence of demographic characteristics and family background on the study results, this study included relevant variables as control variables in the regression equations. Specifically, these include: (1) gender (0 = male, 1 = female) and age (continuous variable); (2) Family structure variables, with a dummy variable to control for whether the family is single-parent (0 = no, 1 = yes); (3) Upbringing environment variables, with a dummy variable to control for whether the individual experienced being a left-behind child (0 = no, 1 = yes). By including these control variables, the objectivity and validity of the research findings are ensured. For full replication, the complete questionnaire is provided in the Appendix.

### Data analysis strategy

Data analysis was conducted using SPSS 26.0 for descriptive statistics, tests of significance, and reliability analysis, and Mplus 8.3 was used for structural equation modeling to test for indirect effects. Since the study involved three dependent variables—cognitive reappraisal, expressive suppression, and difficulty in emotional regulation—the Bonferroni correction was applied to set the significance level at *α* = 0.017 (0.05/3) to control for Type I error rates. The missing data rate for core variables ranged from 0.3 to 1.8%, indicating a low level of missing data. Missing data were handled using Full Information Maximum Likelihood (FIML) estimation in Mplus, which utilizes all available data without requiring imputation. Independent samples *t*-tests were used to compare differences in scores across variables between urban and rural students, with Cohen’s d effect size and its 95% confidence interval reported. To maintain consistency in the sign of the effect size, this study adopted the following convention: when urban freshmen scored higher than rural freshmen, d was negative; when rural freshmen scored higher than urban freshmen, d was positive.

To test for indirect effects, a structural equation model was employed to conduct a multiple mediation analysis, incorporating both psychological fitness and social fitness as parallel mediating variables. To compare the relative importance of each path, this study reports specific indirect effects, total indirect effects, and relative contribution percentages. All effect estimates are statistical associations based on cross-sectional data; no causal inferences are drawn. To test whether there are significant differences in the indirect effects across different mediation pathways, Bootstrap methods were further employed for comparative testing. The models were estimated using maximum likelihood estimation, with chi-square (*χ*^2^), the comparative fit index, the Tucker-Lewis index, the root mean square error of approximation, and the standardized root mean square error of residuals serving as measures of model fit. Control variables were regressed against the independent variable and all mediating variables. Specific indirect effects and total indirect effects are reported, and relative contribution percentages are calculated. To test whether there are significant differences in the indirect effects of different mediation paths, this study further employed the Bootstrap method for comparative testing of indirect effects. The specific settings were as follows: a bias-corrected percentile Bootstrap method, with 5,000 repetitions, to calculate 95% confidence intervals.

## Results

### Descriptive statistics and correlation analysis

This study first conducted descriptive statistics and correlation analyses on the core variables; the results are shown in. The absolute values of skewness for all variables were less than 2, and the absolute values of kurtosis were less than 5, indicating that the data are approximately normally distributed and satisfy the basic assumptions for subsequent parametric tests.

Structural Equation Model Fit Tests: Prior to conducting the multiple mediation analysis, this study first examined the construct validity of the measurement model. The results of confirmatory factor analysis indicated that the model comprising the three dimensions of physical fitness, psychological fitness, and social fitness fit well: *χ*^2^/df = 3.52, CFI = 0.95, TLI = 0.94, RMSEA = 0.055, SRMR = 0.040. The six-factor model of emotional regulation difficulties exhibited good fit indices: *χ*^2^/df = 2.96, CFI = 0.92, TLI = 0.91, RMSEA = 0.062, SRMR = 0.055. All of the above fit indices met acceptable standards, indicating that the measurement models possess good construct validity and are suitable for subsequent structural equation modeling analysis.

**Figure 2:**
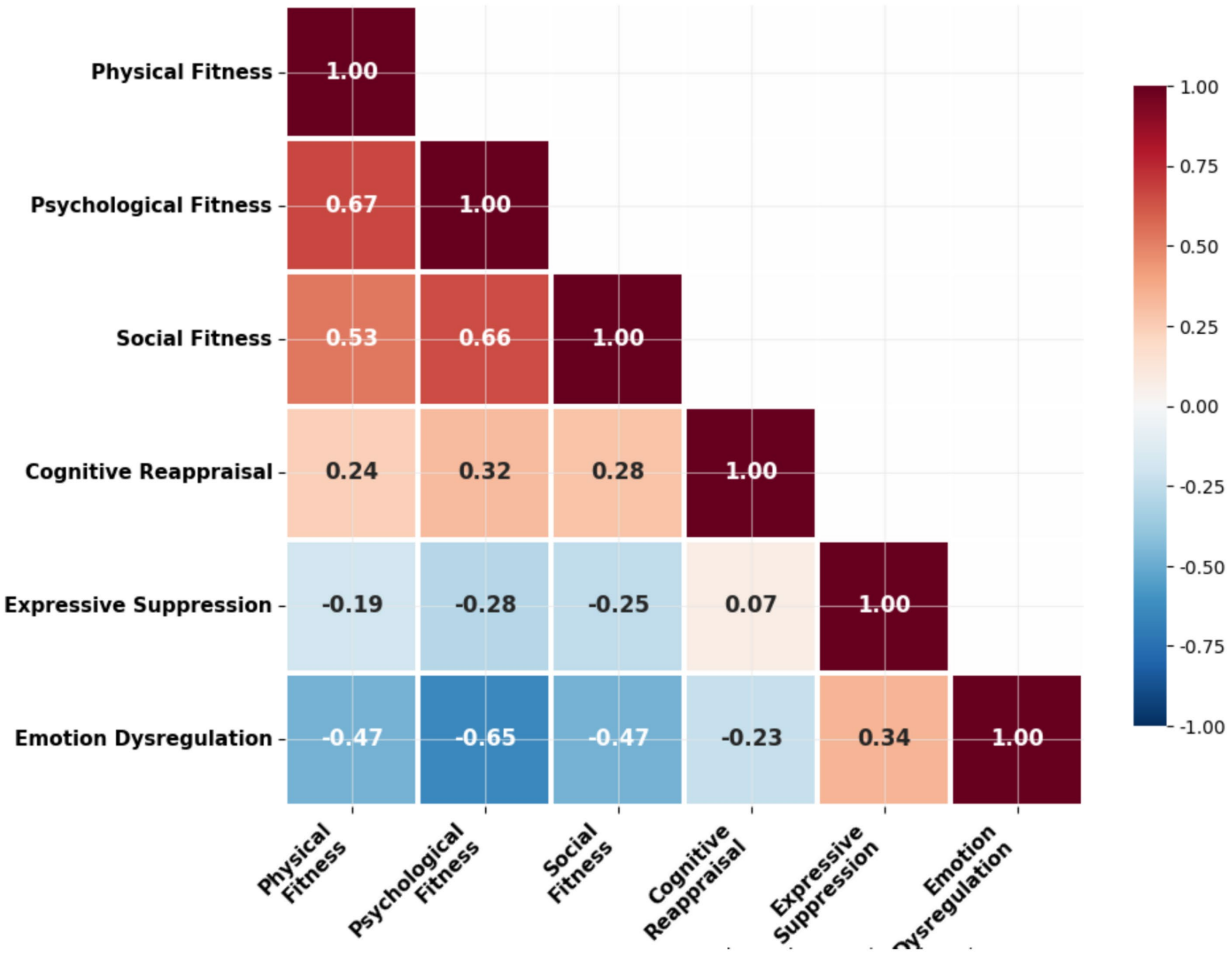
Descriptive statistics and correlation matrix.

The correlation coefficient between psychological adaptability and social adaptability was 0.66 (*p <* 0.001), indicating a moderately high correlation. Examining the relationships between all three health fitness dimensions and emotional regulation indicators separately, significant correlations were found for all three, consistent with theoretical expectations: physical, psychological, and social adaptability were all significantly positively correlated with cognitive reappraisal (r = 0.24, 0.32, 0.28; *p* < 0.001) and significantly negatively correlated with emotional regulation difficulties (r = −0.47, −0.65, −0.47; *p* < 0.001) (Figure 2).

### Test for urban–rural differences

To examine the impact of urban–rural differences on health-related fitness and emotional regulation, this study used an independent samples t-test to compare the differences in scores across various variables between urban and rural freshmen.

In terms of physical fitness, rural freshmen scored significantly higher than urban freshmen (t = −5.32, *p* < 0.001, d = −0.13); In terms of psychological fitness, urban freshmen scored significantly higher than rural freshmen (t = 2.86, *p* = 0.004, d = −0.13, *p* < 0.001); in terms of social fitness, urban freshmen also scored significantly higher than rural freshmen (t = 4.82, *p* < 0.001, d = −0.24). Effect size analysis indicates that the effect sizes for urban–rural differences range from 0.05 to 0.24 in absolute value, ranging from small to medium effect sizes.

In terms of emotional regulation, rural freshmen scored significantly higher on emotional regulation difficulties than urban freshmen (t = −4.82, *p* < 0.001, d = 0.12), while their scores on cognitive reappraisal were significantly lower than those of urban freshmen (t = 2.46, *p* = 0.014, d = −0.06, *p < 0.05). There were no significant differences between the two groups on the expression inhibition dimension (t = −1.89, *p* = 0.058, d = 0.05).

**Figure 3:**
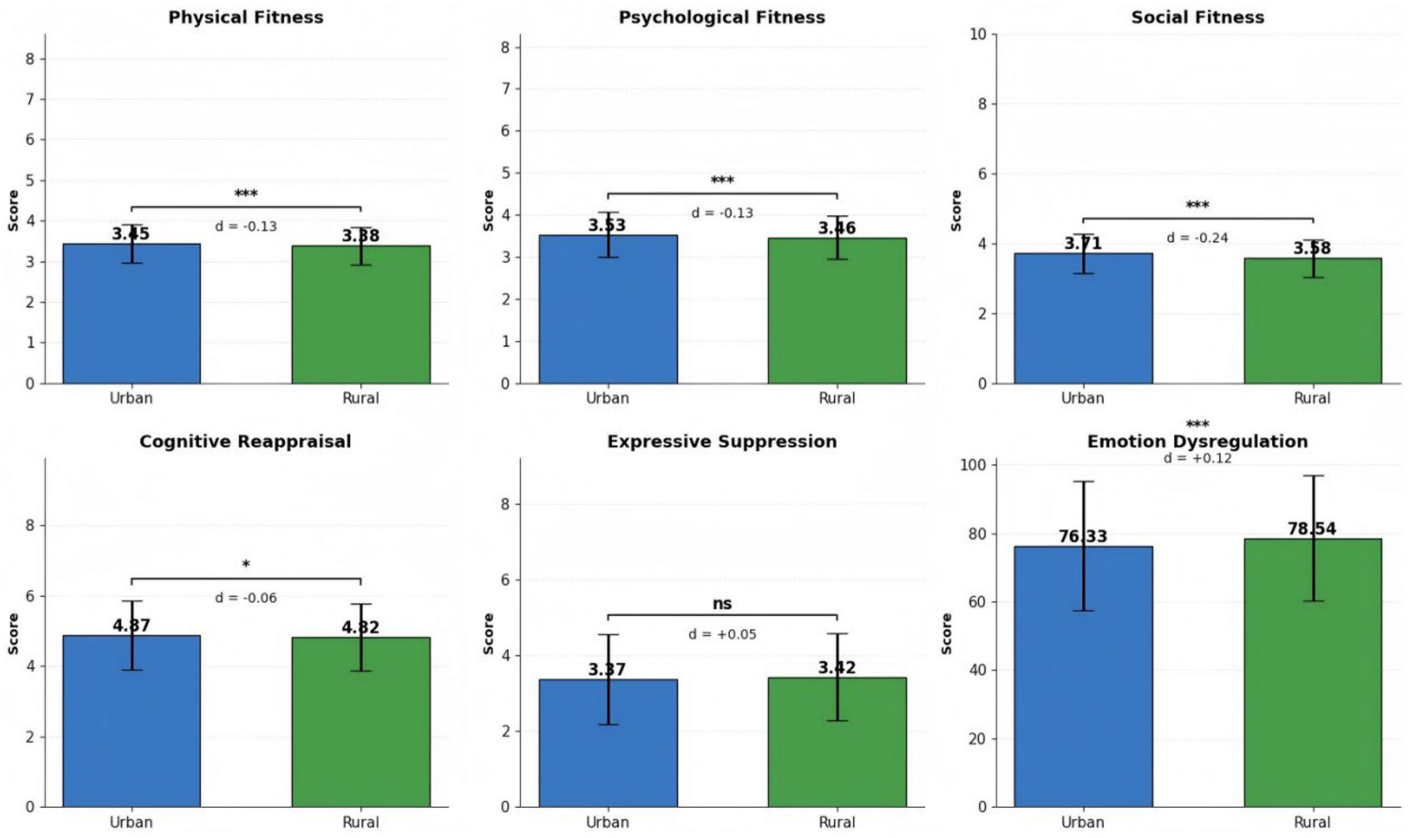
Comparison of urban–rural differences.

Given that single-parent households and experiences of being left behind may be covariates of urban–rural differences, this study further conducted a covariance analysis with emotional regulation difficulties as the dependent variable. The results indicate that, after controlling for single-parent households and experiences of being left behind, the main effect of urban–rural differences remained significant [*F*(1,6712) = 12.38, *p* < 0.001], but the effect size decreased from d = 0.12 to d = 0.07 (Figure 3).

### Indirect effects of psychological and social competence

To examine whether physical, psychological, and social competence exert statistical indirect effects on the relationship between urban–rural differences and emotional regulation, this study constructed a structural equation model (SEM) and included physical fitness, psychological fitness, and social fitness as parallel mediating variables. The model fit results showed: *χ*^2^/df = 3.68, CFI = 0.94, TLI = 0.93, RMSEA = 0.058, and SRMR = 0.045, indicating a good model fit.

**Figure 4:**
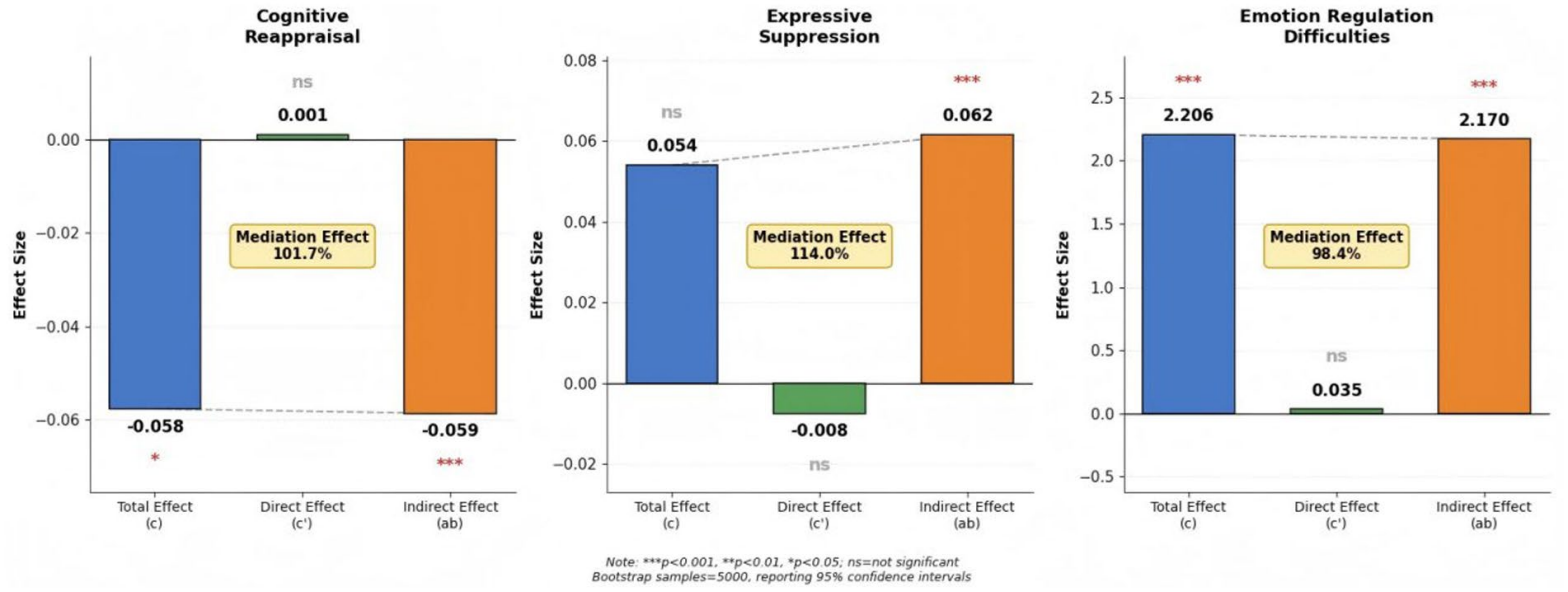
Path diagram of indirect effects on health and fitness.

The model’s coefficient of determination (R^2^) for each endogenous variable was as follows: physical fitness 0.038, psychological adaptability 0.042, social fitness 0.038, difficulty with emotional regulation 0.47, cognitive reappraisal 0.39, and expressive inhibition 0.21; Physical fitness significantly predicted all three emotional regulation outcomes (β = −0.04 to 0.09, *p* < 0.05), though with comparatively smaller effects. All direct path coefficients and their significance levels in the structural model are as follows: Urban–rural differences significantly and negatively predicted psychological fitness (*β* = −0.056, *p* = 0.001) and social fitness (*β* = −0.10, *p* = 0.001); Psychological adaptability significantly and negatively predicted difficulties in emotional regulation (*β* = −0.54, *p* < 0.001), and significantly and positively predicted cognitive reappraisal (*β* = 0.21, *p* < 0.001) and expressive suppression (*β* = −0.19, *p* < 0.001); Social fitness significantly and negatively predicted difficulties in emotional regulation (*β* = −0.06, *p* < 0.001), and significantly and positively predicted cognitive reappraisal (*β* = 0.11, *p* < 0.001) and expressive suppression (*β* = −0.10, *p* < 0.001) (Figure 4).

### Multidimensional relative contributions

**Figure 5:**
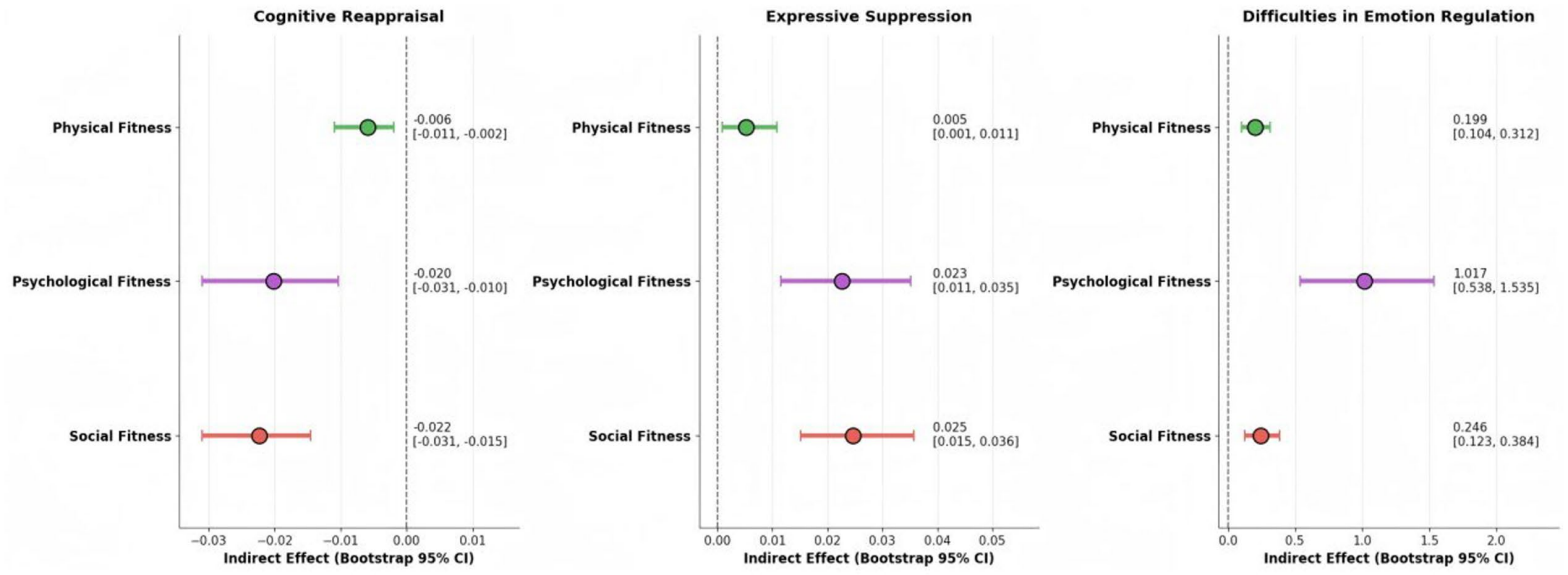
Contribution diagram for each dimension of the multiple mediation model.

To gain a deeper understanding of how different dimensions contribute to physical, psychological, and social adaptability, this study employed structural equation modeling (SEM) to conduct a parallel mediation analysis and used the Bootstrap method to test the statistical significance of the indirect effects (Figure 5).

The results show that there are significant differences in the contribution patterns across the three dimensions:

In the emotional regulation difficulties model, the statistical indirect effect of psychological fitness (*β* = −1.017, 95% CI [0.538, 1.535]) was significantly greater than that of social fitness (*β* = 0.246, 95% CI [0.123, 0.384]) and physical fitness (*β* = 0.199, 95% CI [0.104, 0.312]) and a test of the difference in statistical indirect effects indicated that the difference between the statistical indirect effects of psychological fitness and social fitness was significant (Δ*β* =  0.771, 95% CI [0.328, 1.214]).

In terms of cognitive reassessment, Physical fitness (*β* = −0.006, 95% CI [−0.011, −0.002]), social fitness and psychological fitness contributed approximately equally, with confidence intervals for both excluding 0. A test for differences in statistical indirect effects showed no significant difference between psychological and social fitness.

**Figure 6:**
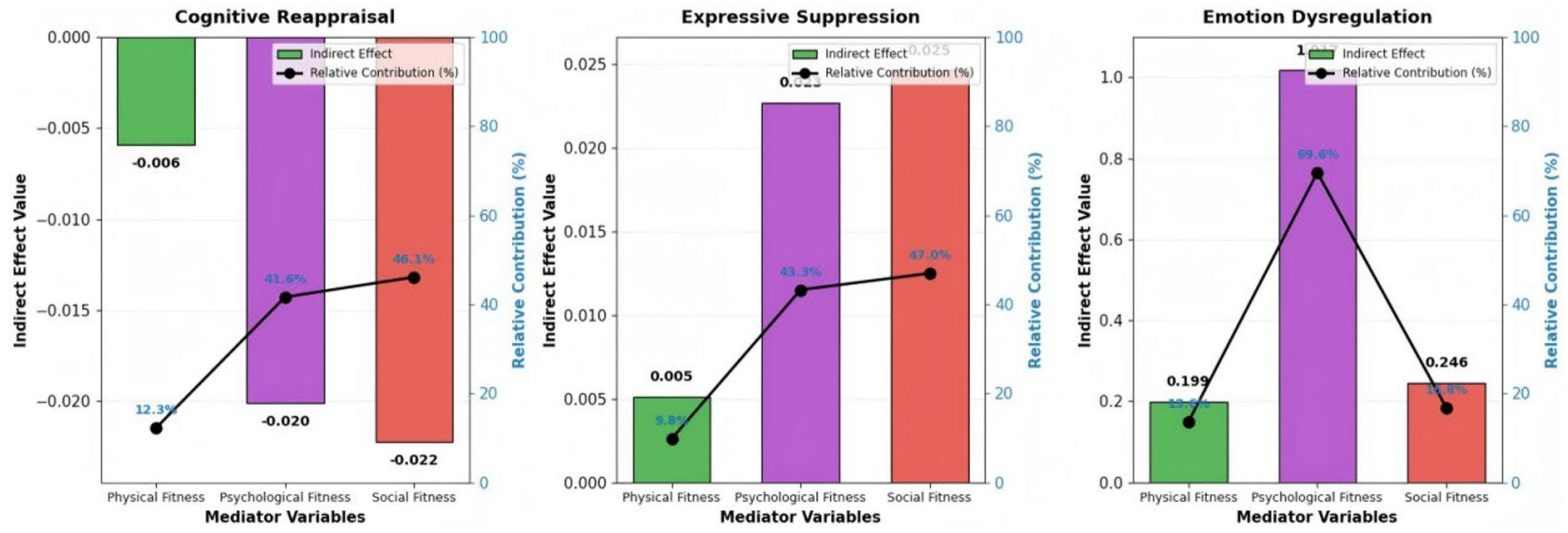
Chart showing the relative percentage contributions of mediating pathways.

The confidence intervals for the indirect effects of expressive suppression on psychological competence and social competence do not include zero; there is no significant difference between the two statistical indirect effects, and the overall indirect effect is positive (Figure 6).

### Robustness tests

To ensure the reliability of the findings, this study conducted a series of robustness tests:

First, the statistical analysis of indirect effects described above was repeated using the scores on the six subscales of the Emotional Regulation Difficulty Scale as the dependent variable instead of the total score. The pattern of results was consistent with that of the main analysis, with physical fitness showing significant indirect effects across all six subscales.

Second, after reanalyzing the data following the exclusion of outliers (*n =* 42) falling outside ±3 standard deviations of each variable, none of the core conclusions changed substantially.

Furthermore, when the interaction term between urban–rural differences and gender was included in the model, the effects of the interaction term on all three dependent variables were insignificant (*p* > 0.20), indicating that the statistical association of urban–rural differences is consistent across genders.

**Figure 7:**
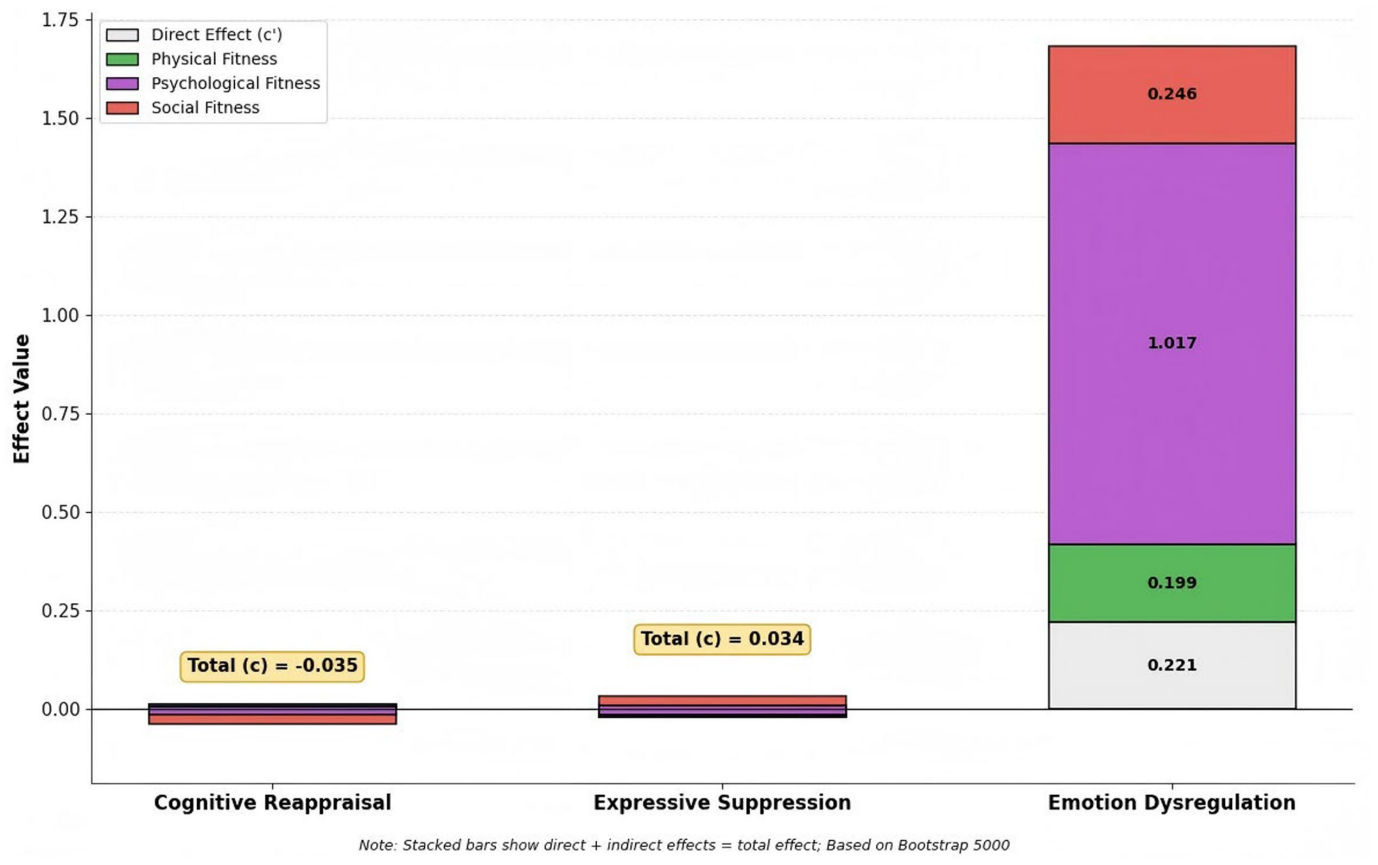
Results of the robustness test.

Finally, the sample was divided into two groups based on left-behind child experience, and multiple mediation analyses were conducted for each group. The results showed that all three mediation paths were significant across both groups, and the Q-test for differences in effect sizes between groups was not significant (*p* = 0.36) (Figure 7).

In summary, the results of all robustness tests are consistent with those of the main analysis, indicating that the core findings of this study remain robust across various conditions, including the operationalization of the dependent variable, the handling of outliers, and the grouping by gender.

## Discussion

### Indirect effects of psychological and social competence and their theoretical explanations

Based on a large sample of 6,718 first-year college students, this study conducted an exploratory analysis of the statistical indirect effects of urban–rural differences, psychological resources, social resources, and emotional regulation, with a particular focus on the statistical indirect effects of psychological and social resources. The results generally support the research hypotheses and reveal relatively clear pathways of resource transmission. Rural freshmen statistically exhibited lower levels of emotional regulation and insufficient psychological and social adaptability resources, whereas urban freshmen demonstrated higher emotional regulation abilities and greater psychological and social adaptability resources; the statistical association between residential area type and individual emotional regulation can be explained through psychological and social adaptability; Psychological resilience was statistically more strongly associated with difficulties in emotional regulation, while social resilience was statistically more strongly associated with cognitive reappraisal and expressive suppression; no significant gender interaction effects were observed. Psychological and social adaptability showed a moderately high correlation (r = 0.66), supporting the notion that these two dimensions possess good discriminant validity and represent distinct yet interrelated concepts. The correlation patterns between both dimensions and emotional regulation indicators align with the theoretical predictions of resource conservation theory, namely that both psychological and social resources exhibit positive statistical associations with adaptive emotional regulation and negative statistical associations with emotional regulation difficulties.

An analysis of urban–rural differences revealed that rural freshmen scored significantly higher than their urban counterparts on measures of emotional regulation difficulties, while their scores on physical, psychological, and social adjustment were all significantly lower than those of urban freshmen. Although the effect sizes were generally small to medium, the differences were statistically consistent and remained significant even after controlling for family background variables such as single-parent households and experiences as left-behind children. This indicates that urban–rural differences have an independent predictive role in freshmen’s psychological adjustment (43). The results of the mediation analysis indicate that psychological fitness and social fitness exert significant statistical mediating effects on the relationship between urban–rural disparities and emotional regulation. When psychological resilience and social resilience were included in the model, the direct statistical predictive effect of urban–rural disparities on emotional regulation indicators was no longer significant, suggesting that urban–rural disparities may be statistically associated with emotional regulation through the mediating variables of psychological resilience and social support. The confidence intervals for the indirect effects do not include 0, indicating that the statistical indirect effects of psychological and social adaptability are significant; the confidence intervals for the direct predictive effects include 0, indicating that after incorporating psychological and social adaptability, the direct statistical predictive effect of urban–rural differences on emotional regulation disappears; The proportion of indirect effects approached or exceeded 100%, further confirming that psychological and social adaptability statistically constitute the transmission pathways linking urban–rural differences to emotional regulation. This result suggests that urban–rural differences in emotional regulation may statistically be mediated through differences in two major resources: psychological resilience and social support. The multiple mediation model further revealed distinct mechanisms of action across the dimensions of health literacy. Psychological health literacy exhibited the strongest statistical indirect association with difficulties in emotional regulation; social health literacy made the greatest statistical indirect contribution to the two emotional regulation strategies of cognitive reappraisal and expressive suppression; in contrast, the independent statistical indirect effect of physical health literacy did not reach statistical significance. These findings suggest that, in explaining urban–rural differences in emotional regulation, psychological and social resources play a more critical role than perceptions of physical fitness.

There is a statistically significant negative correlation between urban–rural disparities and the quantity and quality of resources available to individuals (44). For urban residents, growing up in rural areas is statistically associated with disadvantages in terms of educational capital, social capital, and psychological capital (45). This structural disparity is statistically associated with a lack of advantageous resources available to them when facing challenges after entering college. The resulting negative experiences are statistically negatively correlated with emotional reactions, which in turn are associated with increased adjustment problems. When advantageous resources are insufficient to buffer external stressors, levels of psychological resilience are statistically associated with better coping outcomes. At the same time, differences in living environments are statistically associated with the quantity and types of social resources available to individuals. Although individuals may have similar innate endowments, the external help and support they can access may vary; this difference is statistically associated with an individual’s ability to respond positively to external stimuli (46). It follows that an individual’s psychological processes are statistically correlated with the various subsystems within their environment and the relationships between them. When we view the environment as a dynamic process rather than a static entity, we can observe a statistical correlation between an individual’s development and the interactions between the environmental system and the individual’s internal resources.

In this study, the effect sizes for urban–rural differences (Cohen’s d ranging from 0.05 to 0.24) were below the conventional threshold for a small effect (0.20). While these differences are statistically discernible due to the large sample size, their practical significance should be interpreted with caution. This suggests that urban–rural background alone is a weak predictor of an individual’s emotional regulation ability. However, in the context of public health and from a resource conservation perspective, even small, persistent disparities at the population level can have cumulative effects on mental health service needs. Therefore, the theoretical value of this study lies in elucidating the mediating pathways, rather than in emphasizing the magnitude of the direct urban–rural gap. Researchers and practitioners should avoid labeling students based solely on their urban/rural status and instead focus on assessing and enhancing the proximal psychological and social resources that serve as the actual mechanisms of effect.

This study found that urban–rural psychological differences are not directly caused by place of residence itself, but are statistically associated with differences in the psychological resilience and social support available to individuals. By adopting an integrated theoretical framework of psychological and social fitness, psychological resilience and social support can be unified as capital that individuals draw upon to cope with environmental challenges, thereby more clearly revealing the statistical transmission pathway of “urban–rural differences → resource acquisition → emotional adaptation.” These concepts allow us to intuitively compare, within a single model, that in the statistical prediction of difficulties in emotional regulation, the statistical indirect effect of internal psychological resilience reserves is significantly greater than that of external social support reserves. This direct comparison within the same theoretical framework provides preliminary empirical guidance for prioritizing mental health interventions in higher education institutions. This finding not only deepens our understanding of the mechanisms underlying urban–rural mental health disparities but also offers more concrete entry points for mental health interventions in higher education.

## Explanation of differences in the effects of different dimensions of health and fitness

### The central role of psychological wellbeing in emotional regulation

The results indicate that psychological fitness has the largest statistical indirect effect on difficulties in emotional regulation, suggesting that psychological resilience is a statistically significant internal resource that predicts emotional regulation ability. Psychological fitness reflects an individual’s adaptability and resilience in the face of stress, setbacks, and challenges, with psychological resilience at its core. In the model of emotional regulation difficulties, the statistical indirect effect of psychological fitness was significantly greater than that of social fitness, with the confidence interval of the difference test not including 0. This result indicates that, in the statistical association with emotional regulation difficulties, psychological fitness makes a relatively greater contribution, followed by social fitness.

Psychological resilience exhibited the strongest statistical indirect effect; this finding extends the application of resource conservation theory from total resource volume to resource type. This study suggests that, when facing general adaptive stress, intrinsic psychological resilience may be statistically more strongly associated with emotional regulation than external social support or physiological foundations. Participants with high psychological resilience tend to adopt a more positive attribution style toward negative life events, are more inclined to use cognitive reappraisal and problem-solving strategies to cope with negative events, and are better able to quickly return to baseline levels following exposure to negative emotional stimuli. In contrast, individuals with low psychological resilience are prone to developing negative attribution styles, often dwelling on past misfortunes or struggling to control their anger (47). For rural freshmen who have just entered an unfamiliar environment, psychological resilience serves as their first line of psychological defense against academic and life challenges, while social competence acts as an external buffer system that helps them rebuild a sense of security. The differing contributions of these two factors to various coping strategies provide a detailed picture of the statistical patterns underlying the synergy between internal and external resources. Throughout their upbringing, rural students often face a series of long-term stressors, including insufficient educational resources, low parental income, and unequal development opportunities (48). These stressors are statistically associated with lower levels of psychological resilience, which in turn is statistically associated with greater feelings of discomfort upon entering the new environment of college, and consequently with greater difficulty in effectively regulating emotions when facing setbacks in daily life (49). It is evident that psychological wellbeing serves, to a certain extent, as a key statistical predictor for understanding the urban–rural divide.

### The supportive role of social competence in emotional regulation

Research indicates that, among the emotion regulation strategies of cognitive reappraisal and expressive suppression, social fitness accounts for a larger proportion of the statistical indirect effects than psychological fitness, with the former accounting for a greater share than the latter. Specifically, social wellbeing accounts for 46.1% of the statistical indirect effects in cognitive reappraisal. This suggests that social support is statistically strongly associated with the emotion regulation process (50). In the statistical indirect effects of cognitive reappraisal, physical fitness contributes 12.3%, while, indicating that these two types of resources are nearly equally important in the statistical association with cognitive reappraisal strategies. Social support may provide diverse external perspectives and feedback for cognitive restructuring, while psychological resilience is statistically associated with the internal motivation for individuals to actively engage in cognitive adjustment. Notably, both psychological and social fitness showed small but significant negative associations with expressive suppression. This unexpected direction may reflect the Chinese cultural context, where emotional restraint is sometimes socially adaptive. In collectivistic settings, individuals with higher psychological and social resources may strategically deploy suppression to maintain group harmony, rather than reflecting maladaptive regulation per se. Alternatively, this finding may indicate that resource-rich individuals possess a broader repertoire of regulation strategies, including situation-appropriate suppression.

From a cognitive perspective, social fitness is statistically associated with the complementary function of individual cognition; it provides a variety of information and different perspectives from various sources, and helps prevent cognitive biases resulting from self-focus (51). Following an adverse event, if an individual is unable to receive external feedback, they are prone to overgeneralization and self-deprecation, which can lead to cognitive rigidity. If, at this point, someone provides positive guidance or constructive feedback, it reduces the likelihood of these outcomes and is statistically associated with higher levels of cognitive flexibility (52). From a behavioral perspective, rather than implicitly restructuring their cognition to achieve cognitive reappraisal, people can vent their emotions by seeking understanding and support from others, which serves as an alternative form of expression suppression (53). For those who lack opportunities to interact with others or feel distrustful of others, however, they are forced to resort to more covert methods to cope with negative stimuli; they may tend to adopt expression-suppression strategies to reduce the release of negative emotions. The prominent role of social competence in cognitive reappraisal and expression suppression provides cross-cultural behavioral evidence for relationship regulation theory. Research indicates that social support is not only statistically associated with direct improvements in mood but also serves as a social toolkit for cognitive operations, providing individuals with diverse perspectives for reinterpreting situations. In this sample, the abundance of social support as an external resource was found to have a significant positive statistical association with the use of cognitive reappraisal strategies.

### Reasons for the lack of significant effects on physical fitness

Physical fitness was not included in the final model as a significant statistical indirect path, and this study did not find any clear statistical indirect effects; however, this does not imply that physical health is not statistically associated with emotional regulation. Our study sample consisted of college students, and the assessment tools used were based on subjective evaluations specifically, mental health scales were employed to assess physical health rather than biological factors. Consequently, statistical results may vary due to differences in participants’ psychological states. Overall, first-year college students are in their teenage years, and this age group is generally a relatively healthy population (54). The statistical indirect effect of physical fitness was not significant; rather, it revealed an important caveat: in relatively healthy and homogeneous young populations, the statistical association between physical resources and psychosocial resources may be masked. This suggests that the effects of physical resources may be more pronounced in populations facing genuine physical challenges, thereby defining the scope for future research. Physical health is a complex phenomenon; unlike psychological resilience, it cannot be intuitively observed (55). Therefore, in this study, the predictive role of physical fitness may not be as statistically significant as that of psychological and social fitness.

### Limitations of the study and directions for future research

Robustness tests indicate that the core findings of this study remain consistent across various conditions, including the operationalization of the dependent variable, handling of outliers, and grouping by gender; this enhances the credibility of the results. The limitations of this study are as follows: First, the use of a cross-sectional design prevents the strict establishment of causal relationships between variables. The observed statistical indirect effects of psychological and social adaptability reflect associations with urban–rural differences; causal relationships cannot be established due to the cross-sectional design; however, reverse associations or confounding by third variables may also exist. Second, the sample was drawn from a comprehensive university using convenience sampling, and the proportion of female participants was high (69.8%). As such, it does not represent the characteristics of the national college student population or the widespread educational disparities between urban and rural areas. Third, the use of a single item to measure urban–rural background has inherent limitations. This measurement method reduces urban–rural background to a binary classification, failing to capture its continuity and complexity. This operationalization may lead to an underestimation of urban–rural differences and potentially affect the statistical inferences of the model. Future research should adopt more refined measurement methods to more accurately characterize the relationship between urban–rural background and mental health. Fourth, the Cronbach’s alpha coefficient for the Social Fitness Scale was 0.685, which is at an acceptable threshold; however, the relatively low reliability may introduce measurement error and potentially affect the estimation of the results in this study. In the multiple mediation model, the relatively low reliability of social fitness may weaken its statistical association with emotional regulation indicators, thereby underestimating the estimated indirect effect of the social fitness path and affecting the accuracy of the confidence intervals for the indirect effects. Future research should select or develop social support measurement tools with higher reliability among Chinese university freshmen, or adopt methods to correct for measurement error in structural equation models, in order to more accurately assess the statistical indirect effects of social competence. Fifth, all variables were measured using self-report scales; although the results of the common method bias test were within an acceptable range, issues such as social desirability and recall bias may still exist. Sixth, the effect sizes were small. The effect sizes of urban–rural differences across all variables ranged from 0.05 to 0.24, all of which were smaller than the small effect size threshold (d = 0.20) recommended by Cohen (1988), indicating very small effects. This finding holds significant theoretical implications. In this study, statistical significance must be distinguished from practical significance. The large sample size (*N =* 6,718) allows even minute differences between groups to reach statistical significance, but this does not imply that these differences carry equal clinical or practical significance. Urban and rural backgrounds have very limited explanatory power regarding differences in emotional regulation; researchers should focus more on differences in individual-level psychological and social resources rather than overemphasizing urban–rural classifications. In terms of intervention practice, this suggests that students should not be labeled at risk or targeted for intervention solely based on their urban or rural identity; instead, the assessment and enhancement of proximal resources such as psychological and social competence should be prioritized as more precise intervention targets. The small effect size may also indicate that the impact of urban–rural differences largely depends on specific contextual factors. Future research should test the boundary conditions of this finding in more diverse samples and identify potential moderators that could amplify the effect. Seventh, while this study primarily discusses mediational effects, it has not yet explored potential moderating factors in depth.

In summary, this study can only be considered exploratory in nature and should not be applied in practice until supported by research involving larger samples and a broader range of populations. In future studies, a multicenter sampling approach could be adopted to collect data, with the aim of better determining whether these findings possess generalizability and practical value. Furthermore, building upon a thorough exploration of mediating effects, research should delve deeper into potential moderating factors; this would provide a more profound understanding of the causes underlying the differences in mental health levels between urban and rural students. In future research, a longitudinal study design could be adopted to trace causal sequences, and additional potential moderating variables could be included to further validate and refine the findings of this study.

### Practical implications

Within the sample of this study, this provides effective targets for mental health interventions in higher education institutions. Interventions should not consist of broad, generalized mental health education. Instead, for rural freshmen who generally exhibit higher difficulties in emotional regulation, priority should be given to group training grounded in psychological resilience. The focus should be on building peer support networks and creating a safe social environment, providing students with material and opportunities to practice cognitive restructuring. Activities should be designed to promote self-disclosure and social sharing, allowing students who struggle with self-expression to find healthy outlets as alternatives to repression. However, such intervention recommendations should only be implemented after being supported by further causal evidence. The findings of this study suggest that future psychological assessments for incoming freshmen should incorporate indicators such as physical fitness, psychological resilience, and social support, rather than merely screening for symptoms, thereby shifting the focus from risk warning to resource matching.

## Conclusion

This study, which utilized a large sample of 6,718 first-year college students, identified a statistical indirect association between urban–rural differences and emotional regulation. Compared to urban students, rural students experienced greater difficulty with emotional regulation and lower levels of physical, psychological, and social upon enrollment. Although the effect sizes were small, all findings were statistically significant and consistent across all indicators. Furthermore, physical, psychological, and social fitness served as the statistical mediating pathways through which urban–rural differences influenced emotional regulation. This indicates that the urban–rural differences in emotional regulation do not arise directly but rather result from a statistical indirect association stemming from the differing distributions of physical, psychological, and social fitness between the two groups.

Limitations in physical, psychological, and social fitness in urban and rural environments are among the key factors predicting differences in emotional regulation. Physical, psychological, and social fitness serve as statistical mediating variables in this process, Physical fitness demonstrated significant but comparatively smaller indirect effects. Psychological resilience is the strongest internal predictor of adaptability, capable of predicting performance across the entire emotional regulation difficulty dimension, while social support is the strongest external predictor, capable of predicting performance on the two specific skills of cognitive reappraisal and expressive suppression. The fact that these results hold even after controlling for the statistical effects of single-parent family and left-behind child backgrounds indicates that urban–rural differences have a relatively independent statistical predictive effect rather than being solely determined by family structure. Within the scope of this study’s sample, mental health education programs in higher education institutions could explore focusing on rural students with lower psychological and social adaptability. Efforts should begin with fostering psychological resilience and building social support networks. Pre-enrollment psychological assessments should include items related to health adaptability to facilitate early detection, prevention, and intervention. However, these intervention recommendations should be implemented cautiously, pending further support from causal evidence. This study employs a cross-sectional design; the statistical indirect effects identified reflect only statistical associations between variables and do not allow for inferences of causality. Further validation through longitudinal studies is required in the future. By integrating the bio-psycho-social model with the theory of resource conservation, this study analyzes the issue and proposes—and empirically validates—a statistical explanatory framework that incorporates social contexts, individual resources, and psychological functioning. This framework contributes to our understanding of differences in mental health between urban and rural areas and their statistical predictors.

## Data Availability

The raw data supporting the conclusions of this article will be made available by the authors, without undue reservation.
