# Supplementary material for: The association between urban–rural disparities and emotional regulation abilities among first-year college students: a statistical indirect effects analysis of psychological and social competence
Source: Front Public Health. 2026 Jun 10;14:1836842. doi: 10.3389/fpubh.2026.1836842 (PMC13290984; doi:10.3389/fpubh.2026.1836842)
Supplement: Supplementary file 1 [file Supplementary_file_1.docx]

# Attachment

**College Student Health-Related Fitness Questionnaire**

This study aims to understand the emotional states, prevalence of emotional disorders, and influencing factors among contemporary college students, so as to provide a scientific basis for school mental health education. This questionnaire is anonymous and strictly confidential. Your privacy will not be disclosed to any individual or organization, nor will it affect your academic performance or eligibility for awards and honors. Please complete the questionnaire carefully. Thank you for your support and cooperation!

**1.**Gender [Single Choice]

| Male | Female |
| --- | --- |
| ○ | ○ |

**2.**Are you from a single-parent family? [Single Choice]

| Yes | No |
| --- | --- |
| ○ | ○ |

**3.**Type of residence (urban or rural) [Single Choice]

| Urban | Rural |
| --- | --- |
| ○ | ○ |

**4.**Did you have a left-behind experience (parents working away from home during childhood)? [Single Choice]

| Yes | No |
| --- | --- |
| ○ | ○ |

**5.**When I want to feel more positive emotions (such as joy or happiness), I will change the way I think about the situation. [Single Choice]

| Strongly Disagree | Moderately Disagree | Slightly Disagree | Uncertain | Slightly Agree | Moderately Agree | Strongly Agree |
| --- | --- | --- | --- | --- | --- | --- |
| ○ | ○ | ○ | ○ | ○ | ○ | ○ |

**6.**I do not express my emotions. [Single Choice]

| Strongly Disagree | Moderately Disagree | Slightly Disagree | Uncertain | Slightly Agree | Moderately Agree | Strongly Agree |
| --- | --- | --- | --- | --- | --- | --- |
| ○ | ○ | ○ | ○ | ○ | ○ | ○ |

**7.**When I want to feel less negative emotions (such as sadness or anger), I will change the way I think about the situation. [Single Choice]

| Strongly Disagree | Moderately Disagree | Slightly Disagree | Uncertain | Slightly Agree | Moderately Agree | Strongly Agree |
| --- | --- | --- | --- | --- | --- | --- |
| ○ | ○ | ○ | ○ | ○ | ○ | ○ |

**8.**When I am feeling positive emotions, I am careful not to express them. [Single Choice]

| Strongly Disagree | Moderately Disagree | Slightly Disagree | Uncertain | Slightly Agree | Moderately Agree | Strongly Agree |
| --- | --- | --- | --- | --- | --- | --- |
| ○ | ○ | ○ | ○ | ○ | ○ | ○ |

**9.**When facing a stressful situation, I think about it in a way that helps me stay calm. [Single Choice]

| Strongly Disagree | Moderately Disagree | Slightly Disagree | Uncertain | Slightly Agree | Moderately Agree | Strongly Agree |
| --- | --- | --- | --- | --- | --- | --- |
| ○ | ○ | ○ | ○ | ○ | ○ | ○ |

**10.**I control my emotions by not expressing them. [Single Choice]

| Strongly Disagree | Moderately Disagree | Slightly Disagree | Uncertain | Slightly Agree | Moderately Agree | Strongly Agree |
| --- | --- | --- | --- | --- | --- | --- |
| ○ | ○ | ○ | ○ | ○ | ○ | ○ |

**11.**When I want to feel more positive emotions, I change the way I am thinking about the situation. [Single Choice]

| Strongly Disagree | Moderately Disagree | Slightly Disagree | Uncertain | Slightly Agree | Moderately Agree | Strongly Agree |
| --- | --- | --- | --- | --- | --- | --- |
| ○ | ○ | ○ | ○ | ○ | ○ | ○ |

**12.**I control my emotions by changing the way I think about the situation I am in. [Single Choice]

| Strongly Disagree | Moderately Disagree | Slightly Disagree | Uncertain | Slightly Agree | Moderately Agree | Strongly Agree |
| --- | --- | --- | --- | --- | --- | --- |
| ○ | ○ | ○ | ○ | ○ | ○ | ○ |

**13.**When I am feeling negative emotions, I make sure not to express them. [Single Choice]

| Strongly Disagree | Moderately Disagree | Slightly Disagree | Uncertain | Slightly Agree | Moderately Agree | Strongly Agree |
| --- | --- | --- | --- | --- | --- | --- |
| ○ | ○ | ○ | ○ | ○ | ○ | ○ |

**14.**When I want to feel less negative emotions, I change the way I am thinking about the situation. [Single Choice]

| Strongly Disagree | Moderately Disagree | Slightly Disagree | Uncertain | Slightly Agree | Moderately Agree | Strongly Agree |
| --- | --- | --- | --- | --- | --- | --- |
| ○ | ○ | ○ | ○ | ○ | ○ | ○ |

**15.**When I feel bad (experience unpleasant emotions), I don't know what to do to feel better. [Single Choice]

| Strongly Disagree | Disagree | Somewhat Disagree | Uncertain | Somewhat Agree | Agree | Strongly Agree |
| --- | --- | --- | --- | --- | --- | --- |
| ○ | ○ | ○ | ○ | ○ | ○ | ○ |

**16.**When I feel bad, I feel helpless to change the way I feel. [Single Choice]

| Strongly Disagree | Disagree | Somewhat Disagree | Uncertain | Somewhat Agree | Agree | Strongly Agree |
| --- | --- | --- | --- | --- | --- | --- |
| ○ | ○ | ○ | ○ | ○ | ○ | ○ |

**17.**When I feel bad, I lack effective strategies for eliminating such feelings (e.g., specific activities or techniques). [Single Choice]

| Strongly Disagree | Disagree | Somewhat Disagree | Uncertain | Somewhat Agree | Agree | Strongly Agree |
| --- | --- | --- | --- | --- | --- | --- |
| ○ | ○ | ○ | ○ | ○ | ○ | ○ |

**18.**When I feel bad, I have no control over the intensity and duration of these emotions. [Single Choice]

| Strongly Disagree | Disagree | Somewhat Disagree | Uncertain | Somewhat Agree | Agree | Strongly Agree |
| --- | --- | --- | --- | --- | --- | --- |
| ○ | ○ | ○ | ○ | ○ | ○ | ○ |

**19.**When I feel bad, these emotions interfere with my ability to get work done. [Single Choice]

| Strongly Disagree | Disagree | Somewhat Disagree | Uncertain | Somewhat Agree | Agree | Strongly Agree |
| --- | --- | --- | --- | --- | --- | --- |
| ○ | ○ | ○ | ○ | ○ | ○ | ○ |

**20.**When I feel bad, I am unable to carry out tasks that I should be doing. [Single Choice]

| Strongly Disagree | Disagree | Somewhat Disagree | Uncertain | Somewhat Agree | Agree | Strongly Agree |
| --- | --- | --- | --- | --- | --- | --- |
| ○ | ○ | ○ | ○ | ○ | ○ | ○ |

**21.**When I feel bad, I have difficulty motivating myself to deal with important matters (work, household chores, academics, etc.). [Single Choice]

| Strongly Disagree | Disagree | Somewhat Disagree | Uncertain | Somewhat Agree | Agree | Strongly Agree |
| --- | --- | --- | --- | --- | --- | --- |
| ○ | ○ | ○ | ○ | ○ | ○ | ○ |

**22.**When I feel bad, everything feels difficult. [Single Choice]

| Strongly Disagree | Disagree | Somewhat Disagree | Uncertain | Somewhat Agree | Agree | Strongly Agree |
| --- | --- | --- | --- | --- | --- | --- |
| ○ | ○ | ○ | ○ | ○ | ○ | ○ |

**23.**When I feel bad, I engage in foolish behaviors. [Single Choice]

| Strongly Disagree | Disagree | Somewhat Disagree | Uncertain | Somewhat Agree | Agree | Strongly Agree |
| --- | --- | --- | --- | --- | --- | --- |
| ○ | ○ | ○ | ○ | ○ | ○ | ○ |

**24.**When I feel bad, my behavior becomes out of control. [Single Choice]

| Strongly Disagree | Disagree | Somewhat Disagree | Uncertain | Somewhat Agree | Agree | Strongly Agree |
| --- | --- | --- | --- | --- | --- | --- |
| ○ | ○ | ○ | ○ | ○ | ○ | ○ |

**25.**When I feel bad, I have difficulty controlling my actions. [Single Choice]

| Strongly Disagree | Disagree | Somewhat Disagree | Uncertain | Somewhat Agree | Agree | Strongly Agree |
| --- | --- | --- | --- | --- | --- | --- |
| ○ | ○ | ○ | ○ | ○ | ○ | ○ |

**26.**When I feel bad, I experience strong urges to take risks. [Single Choice]

| Strongly Disagree | Disagree | Somewhat Disagree | Uncertain | Somewhat Agree | Agree | Strongly Agree |
| --- | --- | --- | --- | --- | --- | --- |
| ○ | ○ | ○ | ○ | ○ | ○ | ○ |

**27.**When I feel bad, I believe these feelings must be eliminated at all costs. [Single Choice]

| Strongly Disagree | Disagree | Somewhat Disagree | Uncertain | Somewhat Agree | Agree | Strongly Agree |
| --- | --- | --- | --- | --- | --- | --- |
| ○ | ○ | ○ | ○ | ○ | ○ | ○ |

**28.**When I feel bad, I cannot tolerate these emotions. [Single Choice]

| Strongly Disagree | Disagree | Somewhat Disagree | Uncertain | Somewhat Agree | Agree | Strongly Agree |
| --- | --- | --- | --- | --- | --- | --- |
| ○ | ○ | ○ | ○ | ○ | ○ | ○ |

**29.**When I feel bad, I always try to completely eliminate these feelings. [Single Choice]

| Strongly Disagree | Disagree | Somewhat Disagree | Uncertain | Somewhat Agree | Agree | Strongly Agree |
| --- | --- | --- | --- | --- | --- | --- |
| ○ | ○ | ○ | ○ | ○ | ○ | ○ |

**30.**When I feel bad, I believe these emotions are unacceptable. [Single Choice]

| Strongly Disagree | Disagree | Somewhat Disagree | Uncertain | Somewhat Agree | Agree | Strongly Agree |
| --- | --- | --- | --- | --- | --- | --- |
| ○ | ○ | ○ | ○ | ○ | ○ | ○ |

**31.**Do you think your body figure is well-proportioned? [Single Choice]

| Very Disproportionate | Disproportionate | Average | Well-Proportioned | Very Well-Proportioned |
| --- | --- | --- | --- | --- |
| ○ | ○ | ○ | ○ | ○ |

**32.**How is your eyesight? [Single Choice]

| Very Poor | Poor | Average | Good | Very Good |
| --- | --- | --- | --- | --- |
| ○ | ○ | ○ | ○ | ○ |

**33.**How is your hearing? [Single Choice]

| Very Poor | Poor | Average | Good | Very Good |
| --- | --- | --- | --- | --- |
| ○ | ○ | ○ | ○ | ○ |

**34.**Do you have any head discomfort? (e.g., dizziness, headache, heaviness in the head, head distension, scalp numbness, etc.) [Single Choice]

| Always | Often | Sometimes | Rarely | Never |
| --- | --- | --- | --- | --- |
| ○ | ○ | ○ | ○ | ○ |

**35.**When at rest, do you experience palpitations or heart palpitations? [Single Choice]

| Always | Often | Sometimes | Rarely | Never |
| --- | --- | --- | --- | --- |
| ○ | ○ | ○ | ○ | ○ |

**36.**Do you have difficulty climbing three to five flights of stairs? [Single Choice]

| Very Easy | Relatively Easy | Average | Relatively Difficult | Very Difficult |
| --- | --- | --- | --- | --- |
| ○ | ○ | ○ | ○ | ○ |

**37.**Do you have difficulty walking one kilometer? [Single Choice]

| Very Easy | Relatively Easy | Average | Relatively Difficult | Very Difficult |
| --- | --- | --- | --- | --- |
| ○ | ○ | ○ | ○ | ○ |

**38.**Do you have difficulty bending over to touch your toes with your hands without bending your knees? [Single Choice]

| Very Easy | Relatively Easy | Average | Relatively Difficult | Very Difficult |
| --- | --- | --- | --- | --- |
| ○ | ○ | ○ | ○ | ○ |

**39.**Do you have difficulty performing daily household chores? [Single Choice]

| Very Easy | Relatively Easy | Average | Relatively Difficult | Very Difficult |
| --- | --- | --- | --- | --- |
| ○ | ○ | ○ | ○ | ○ |

**40.**Do you have difficulty participating in high-energy-expenditure activities (e.g., strenuous physical exercise, moving heavy objects, etc.)? [Single Choice]

| Very Easy | Relatively Easy | Average | Relatively Difficult | Very Difficult |
| --- | --- | --- | --- | --- |
| ○ | ○ | ○ | ○ | ○ |

**41.**Are you susceptible to illness when seasons change? (e.g., colds, skin allergies, etc.) [Single Choice]

| Very Unlikely | Relatively Unlikely | Average | Relatively Likely | Very Likely |
| --- | --- | --- | --- | --- |
| ○ | ○ | ○ | ○ | ○ |

**42.**Do you think external disturbances (e.g., noise, light, etc.) greatly affect your rest and work? [Single Choice]

| No Effect | Slight Effect | Average | Relatively Large Effect | Very Large Effect |
| --- | --- | --- | --- | --- |
| ○ | ○ | ○ | ○ | ○ |

**43.**Do you recover easily from a cold? [Single Choice]

| Very Easy | Relatively Easy | Average | Relatively Difficult | Very Difficult |
| --- | --- | --- | --- | --- |
| ○ | ○ | ○ | ○ | ○ |

**44.**When you feel unwell (e.g., fatigue, dizziness, headache, etc.), can you get relief after a day's rest? [Single Choice]

| Completely | Most of the Time | Sometimes | Rarely | Not at All |
| --- | --- | --- | --- | --- |
| ○ | ○ | ○ | ○ | ○ |

**45.**Compared with your peers, how do you rate your physical fitness (physical/bodily adaptability)? [Single Choice]

| Very Poor | Poor | Average | Good | Very Good |
| --- | --- | --- | --- | --- |
| ○ | ○ | ○ | ○ | ○ |

**46.**Can you concentrate on one thing easily? [Single Choice]

| Very Unlikely | Relatively Unlikely | Average | Relatively Likely | Very Likely |
| --- | --- | --- | --- | --- |
| ○ | ○ | ○ | ○ | ○ |

**47.**Compared with before, how do you feel about your memory? [Single Choice]

| Very Poor | Poor | Average | Good | Very Good |
| --- | --- | --- | --- | --- |
| ○ | ○ | ○ | ○ | ○ |

**48.**Do you believe that through your own efforts, you can achieve your predetermined goals? [Single Choice]

| Completely Believe | Relatively Believe | Average | Relatively Disbelieve | Do Not Believe at All |
| --- | --- | --- | --- | --- |
| ○ | ○ | ○ | ○ | ○ |

**49.**When your predetermined goal seems difficult to achieve, do you give up easily? [Single Choice]

| Never | Rarely | Sometimes | Often | Always |
| --- | --- | --- | --- | --- |
| ○ | ○ | ○ | ○ | ○ |

**50.**Are you hopeful about your future? [Single Choice]

| Very Hopeful | Relatively Hopeful | Average | Less Hopeful | No Hope |
| --- | --- | --- | --- | --- |
| ○ | ○ | ○ | ○ | ○ |

**51.**Do you get discouraged by failure? [Single Choice]

| Never | Rarely | Sometimes | Often | Always |
| --- | --- | --- | --- | --- |
| ○ | ○ | ○ | ○ | ○ |

**52.**Do you feel lonely? [Single Choice]

| Never | Rarely | Sometimes | Often | Always |
| --- | --- | --- | --- | --- |
| ○ | ○ | ○ | ○ | ○ |

**53.**Do you feel afraid for no reason? [Single Choice]

| Never | Rarely | Sometimes | Often | Always |
| --- | --- | --- | --- | --- |
| ○ | ○ | ○ | ○ | ○ |

**54.**Do you feel depressed or in low spirits? [Single Choice]

| Not at All | Rarely | Sometimes | Often | Always |
| --- | --- | --- | --- | --- |
| ○ | ○ | ○ | ○ | ○ |

**55.**Do you feel restless or fidgety? [Single Choice]

| Not at All | Rarely | Sometimes | Often | Always |
| --- | --- | --- | --- | --- |
| ○ | ○ | ○ | ○ | ○ |

**56.**Do you feel mentally tense? [Single Choice]

| Not at All | Rarely | Sometimes | Often | Always |
| --- | --- | --- | --- | --- |
| ○ | ○ | ○ | ○ | ○ |

**57.**Compared with your peers, how do you rate your psychological fitness (psychological adaptability)? [Single Choice]

| Very Poor | Poor | Average | Good | Very Good |
| --- | --- | --- | --- | --- |
| ○ | ○ | ○ | ○ | ○ |

**58.**Is your family life harmonious? [Single Choice]

| Very Disharmonious | Relatively Disharmonious | Average | Relatively Harmonious | Very Harmonious |
| --- | --- | --- | --- | --- |
| ○ | ○ | ○ | ○ | ○ |

**59.**Regarding unpleasant events in your life, studies, and work, can you handle them properly? [Single Choice]

| Not at All | Rarely | Sometimes | Most of the Time | Completely |
| --- | --- | --- | --- | --- |
| ○ | ○ | ○ | ○ | ○ |

**60.**Can you adapt to new living, learning, and working environments relatively quickly? [Single Choice]

| Not at All | Rarely | Sometimes | Most of the Time | Completely |
| --- | --- | --- | --- | --- |
| ○ | ○ | ○ | ○ | ○ |

**61.**Overall, how do you evaluate the role you play in your work, studies, and life? [Single Choice]

| Very Incompetent | Relatively Incompetent | Average | Relatively Competent | Very Competent |
| --- | --- | --- | --- | --- |
| ○ | ○ | ○ | ○ | ○ |

**62.**Do you maintain regular contact with relatives and friends (e.g., visiting each other, phone greetings, correspondence, etc.)? [Single Choice]

| Never Contact | Rarely Contact | Sometimes Contact | Often Contact | Very Frequently |
| --- | --- | --- | --- | --- |
| ○ | ○ | ○ | ○ | ○ |

**63.**When you need help, do family members, colleagues, or friends provide you with material or emotional support and assistance? [Single Choice]

| Never | Rarely | Sometimes | Often | Always |
| --- | --- | --- | --- | --- |
| ○ | ○ | ○ | ○ | ○ |

**64.**When encountering difficulties, do you actively seek support and help from others? [Single Choice]

| Never | Rarely | Sometimes | Often | Always |
| --- | --- | --- | --- | --- |
| ○ | ○ | ○ | ○ | ○ |

**65.**Do you have friends with whom you can share joy and sorrow? [Single Choice]

| None at All | Relatively Few | Average | Relatively Many | A Great Many (more than five) |
| --- | --- | --- | --- | --- |
| ○ | ○ | ○ | ○ | ○ |

**66.**Do you have many close colleagues, classmates, neighbors, relatives, or friends? [Single Choice]

| None at All | Relatively Few | Average | Relatively Many | A Great Many (more than five) |
| --- | --- | --- | --- | --- |
| ○ | ○ | ○ | ○ | ○ |

**67.**Compared with your peers, how do you rate your social fitness (social adaptability)? [Single Choice]

| Very Poor | Poor | Average | Good | Very Good |
| --- | --- | --- | --- | --- |
| ○ | ○ | ○ | ○ | ○ |

**68.**Compared with your peers, how do you rate your health-related fitness (overall adaptability in physical, psychological, and social aspects)? [Single Choice]

| Very Poor | Poor | Average | Good | Very Good |
| --- | --- | --- | --- | --- |
| ○ | ○ | ○ | ○ | ○ |
